# Adaptive Gene Content and Allele Distribution Variations in the Wild and Domesticated Populations of *Saccharomyces cerevisiae*

**DOI:** 10.3389/fmicb.2021.631250

**Published:** 2021-02-17

**Authors:** Da-Yong Han, Pei-Jie Han, Karl Rumbold, Anbessa Dabassa Koricha, Shou-Fu Duan, Liang Song, Jun-Yan Shi, Kuan Li, Qi-Ming Wang, Feng-Yan Bai

**Affiliations:** ^1^State Key Laboratory of Mycology, Institute of Microbiology, Chinese Academy of Sciences, Beijing, China; ^2^College of Life Sciences, University of Chinese Academy of Sciences, Beijing, China; ^3^School of Molecular and Cell Biology, University of the Witwatersrand, Johannesburg, South Africa; ^4^Department of Biology, College of Natural Sciences, Jimma University, Jimma, Ethiopia

**Keywords:** *Saccharomyces cerevisiae*, domestication, population genomics, adaptive evolution, homing endonuclease VDE

## Abstract

Recent studies on population genomics of *Saccharomyces cerevisiae* have substantially improved our understanding of the genetic diversity and domestication history of the yeast. However, the origin of the domesticated population of *S. cerevisiae* and the genomic changes responsible for ecological adaption of different populations and lineages remain to be fully revealed. Here we sequenced 64 African strains from various indigenous fermented foods and forests in different African countries and performed a population genomic analysis on them combined with a set of previously sequenced worldwide *S. cerevisiae* strains representing the maximum genetic diversity of the species documented so far. The result supports the previous observations that the wild and domesticated populations of *S. cerevisiae* are clearly separated and that the domesticated population diverges into two distinct groups associated with solid- and liquid-state fermentations from a single ancestor. African strains are mostly located in basal lineages of the two domesticated groups, implying a long domestication history of yeast in Africa. We identified genes that mainly or exclusively occur in specific groups or lineages and genes that exhibit evident group or lineage specific allele distribution patterns. Notably, we show that the homing endonuclease VDE is generally absent in the wild but commonly present in the domesticated lineages of *S. cerevisiae*. The genes with group specific allele distribution patterns are mostly enriched in functionally similar or related fundamental metabolism processes, including the evolutionary conserved TOR signaling pathway.

## Introduction

The budding yeast *Saccharomyces cerevisiae* is used worldwide in baking and alcoholic beverage production and the earliest evidence for wine-like beverage fermentation dates back to Neolithic times about 9,000 years ago (McGovern et al., 2004). *S. cerevisiae* is also the most extensively studied eukaryote as a model in physiology, genetics, and cellular and molecular biology. *S. cerevisiae* was once considered a domesticated species with distribution limited in man-made environments (Martini, 1993; Vaughanmartini and Martini, 1995; Naumov, 1996; Camperio-Ciani et al., 2004). Recent studies have shown that *S. cerevisiae* widely distributes in nature and occurs in highly diversified substrates from human-associated environments as well as habitats remote from human activity (e.g., primeval forests) (Wang et al., 2012). Thus, the species has been becoming a powerful system in the studies of population and evolution genomics, ecology and biogeography. The genomes of more than 2,500 *S. cerevisiae* strains have been sequenced independently by different laboratories in the world (Libkind et al., 2020), aiming to illuminate the natural and domestication histories of the yeast and evolutionary forces shaping its biodiversity. Population genetics and genomics studies have shown that the wild and domesticated populations of *S. cerevisiae* are clearly separated. The genetic diversity of the species is mainly contributed by the wild lineages found in China or Far East Asia (Wang et al., 2012; Liti, 2015; Duan et al., 2018). This area also harbors the oldest lineages of the species documented so far. Therefore, an out-of-China origin hypothesis of *S. cerevisiae* has been proposed (Wang et al., 2012; Duan et al., 2018; Peter et al., 2018). However, it remains unclear whether *S. cerevisiae* was first domesticated in Asia and the domesticated strains were later introduced to other continents, or whether wild *S. cerevisiae* immigrated from Asia to other continents and was then domesticated independently in different places (Steensels et al., 2019). Liti et al. (2009) and Peter et al. (2018) showed close relationships of different domesticated lineages with different wild relatives and thus suggested that multiple independent domestication events lead to the origin of different domesticated lineages of *S. cerevisiae*. Separate domestication events have also been proposed in previous studies based on different strain and data sets (Fay and Benavides, 2005; Legras et al., 2007; Ezeronye and Legras, 2009; Liti et al., 2009; Schacherer et al., 2009; Goddard et al., 2010; Pontes et al., 2020). Specifically, Almeida et al. (2015) showed that the European Wine lineage originated from the Mediterranean oak (MO) lineage. On the other hand, our recent study showed that the domesticated lineages documented worldwide so far share a common ancestor which was likely formed by outcrossing between diverse wild isolates, implying a single domestication event scenario (Duan et al., 2018).

Specific wild and domesticated lineages of *S. cerevisiae* have been recognized in different studies employing strains with different origins and sample sizes (Liti et al., 2009; Wang et al., 2012; Gallone et al., 2016; Duan et al., 2018; Peter et al., 2018; Pontes et al., 2020). The fine phylogenetic relationships among the lineages recognized in different studies remain to be resolved. For example, the CHN-IX lineage containing strains from a subtropical primeval forest located in central China was recognized as the oldest lineage of *S. cerevisiae* in Duan et al. (2018), while a lineage represented by a few strains from Taiwan was resolved as the most basal lineage of the species in Peter et al. (2018). It is unclear if the two lineages belong to a same lineage or, if not, which is older. A specific lineage of *S. cerevisiae* from fermented milk products collected in west China and Mongolia was identified in Duan et al. (2018). A distinct lineage from French dairy products was also identified in Legras et al. (2018) and Peter et al. (2018). It is uncertain whether the Asian and European milk strains belong to a single lineage or share a recent common ancestor due to the same or similar niche.

We have shown that all the domesticated lineages belong to two major monophyletic groups associated with liquid- and solid-state fermentations, respectively (Duan et al., 2018). The liquid-state fermentation (LSF) group is mainly consisted of European industrial strains and the solid-state fermentation (SSF) group contains strains mainly from Asia. Thus, the two groups are also called the European and Asian clades, respectively (Steensels et al., 2019). However, the separation of the two domesticated groups were not recognized in other studies on population genetics and genomics of *S. cerevisiae* (Fay and Benavides, 2005; Liti et al., 2009; Schacherer et al., 2009; Cromie et al., 2013; Strope et al., 2015; Gallone et al., 2016; Legras et al., 2018; Peter et al., 2018; Pontes et al., 2020). It is uncertain if worldwide domesticated strains can simply be assigned to the two groups and if the separation of the two groups is primarily caused by geography or ecology. Addition of more strains associated with indigenous fermented foods from other continents, especially Africa, will be helpful to resolve the problems. Africa is rich of fermented foods and has a long history of fermented food production (Ashenafi, 2008; Koricha et al., 2020). Indeed, an African origin hypothesis of domesticated yeast was proposed in Fay and Benavides (2005). Distinct lineages associated with cocoa and palm wine fermentation in West Africa have been identified (Cromie et al., 2013; Tapsoba et al., 2015; Ludlow et al., 2016; Duan et al., 2018; Peter et al., 2018). However, African populations of *S. cerevisiae* were poorly represented in previous studies in terms of strain numbers and geographic and ecological origins. The diversity and evolution of both wild and domesticated *S. cerevisiae* in Africa remain largely unknown.

In recent years, we isolated a set of *S. cerevisiae* strains from indigenous fermented foods and forests in different African countries and sequenced their genomes. We also sequenced more domesticated *S. cerevisiae* strains associated with solid-state fermentation in China. In this study, we combined the newly sequenced *S. cerevisiae* strains with a set of previously sequenced strains representing the maximum genetic diversity and all lineages of the species documented so far in the world. Our integrated phylogenomic and population genomic analyses reveal new distinct lineages from Africa which are mostly located in basal domesticated lineages. We confirm the separation of the wild and domesticated population and the divergence of the LSF and SSF groups of the domesticated population from a single ancestor. We find the correlation of gene content variation with population, group, and lineage delimitation and identified genes that show group or lineage specific patterns in terms of content or allele distribution. The results provide new insights into the domestication history of *S. cerevisiae*.

## Materials and Methods

### *S. cerevisiae* Isolates

A total of 126 *S. cerevisiae* isolates were sequenced in this study, including 64 isolates from different African countries, 52 isolates associated with Baijiu (Chinese liquor) and ten isolates associated with Huangjiu (rice wine) fermentation from different regions in China (Supplementary Table 1). The genome sequence data from a total of 486 isolates sequenced in other studies (Gallone et al., 2016; Duan et al., 2018; Peter et al., 2018) are included. These isolates represent different lineages of *S. cerevisiae* with different ecological and geographic origins (Supplementary Table 1). The newly sequenced isolates were isolated using either the dilution plating method from fermented food and beverage samples as described in Duan et al. (2018) or the enrichment method from natural samples as described in Wang et al. (2012). Yeast isolates were identified as described in Wang et al. (2012).

### Genome Resequencing, Assembly, and Annotation

The genome DNA of each isolate was extracted using a standard Zymolyase protocol (Amberg et al., 2005). For the isolates sequenced in this study, a paired-end library with an average insert size of 300 bp was prepared and was sequenced using the Illumina Hiseq 2000 platform with 2 × 150 bp reads. The sequence coverages ranged from 109× to 242× (average = 184×; median = 187×). For each library, low-quality and ambiguous reads were trimmed using Trimmomatic (v0.30) (Bolger et al., 2014). The program SPAdes (v3.10.0) (Bankevich et al., 2012) was used to assemble clean reads with *K* = 21, 33, 55, and 77, and the best assembly strategy was selected automatically. AUGUSTUS (v2.5.5) (Stanke et al., 2004) was employed for gene prediction from the final assemblies generated by PAGIT with *S. cerevisiae* S288c as the model using the following parameters (genemodel = complete, protein = on). Then, the BLAST (Altschul et al., 1990) program was used to annotate the gene function through searching for homologous sequences in GenBank.

### Reference-Based Alignment and Variant Calling

The clean paired reads obtained were mapped to the S288c (R64-1-1) genome using the BWA (Li and Durbin, 2009) program with default settings. SAMTools (v1.361) (Li, 2011) was employed to convert the alignment results into the BAM format, and Picard Tools (v1.56)^1^ was used to sort the reads and remove duplicated sequences. VarScan (v2.3.9) (Koboldt et al., 2012) was applied for extracting the variant bases with following parameters (min-coverage = 15, min-avg-qual = 25, min-var-freq = 0.2). In addition, the Genome Analysis Toolkit (GATK v2.7.2) (McKenna et al., 2010) program was used to detect the variable sites, with the ‘stand_call_conf’ and the ‘stand_emit_conf’ were set to 40.0 and 30.0, respectively. The high-quality SNPs extracted were the consistent variation sites obtained from VarScan and GATK. The variation sites with a coverage depth ≥ 15 were remained for subsequent analyses and final SNP extraction. The variation sites of an isolates with a coverage depth greater than four times of the sequence depth of the isolate were probably resulted from sequencing errors or duplicate sequences and thus were removed according to Lam et al. (2010). The variation sites were kept only when at least 80% of the reads were positive for homogeneous sites and at least 20% of the reads were positive for heterogeneous sites.

Among the 486 isolates sequenced in previous studies, variation sites were obtained by the following approaches: SNPs of 244 isolates sequenced in Peter et al. (2018) were extracted from the published Matrix.gvcf.gz file; SNPs of 191 isolates selected from Duan et al. (2018) were offered by the author; SNPs of the rest 51 isolates sequenced in Gallone et al. (2016) were extracted from the assembled genome files deposited in GenBank using show-snps in the MUMmer software (Kurtz et al., 2004) with default setting. A total of 1,537,415 SNPs were obtained from the 612 isolates compared. We then filtered out the sites missing in more than 1% isolates using the tirmAl (Capella-Gutierrez et al., 2009) software and finally obtained a set of 1,382,078 SNPs across all isolates employed.

### Phylogenomics, Structure, and Population Genetics Analyses

Phylogenetic trees were constructed based on the whole genome SNPs, including both homozygous and heterozygous sites. The sequence alignment was subjected to maximum likelihood analysis using the FastTree program (Price et al., 2009). The repeated random haplotype sampling (RRHS) strategy with 100 repetitions was applied as described in Lischer et al. (2014). The 100 ML trees generated were then summarized in a majority rule consensus tree with mean branch lengths and bootstrap values using the SumTrees program (Sukumaran and Holder, 2010). ADMIXTURE (v1.23) (Alexander et al., 2009) was used to detect and quantify the number of populations and the degree of admixture in all 612 isolates. The set of 1,382,078 biallelic segregating sites identified above was filtered further by removing the SNPs with a minor allele frequency (MAF) < 0.01 and the SNPs in linkage-disequilibrium, using PLINK (v1.07) (Purcell et al., 2007) with a window size of 50 SNPs advanced by 5 SNPs at a time and an r2 threshold of 0.5 described in Gallone et al. (2016). ADMIXTURE was run on a filtered set of 239,507 segregating sites, the best-fit K value from 2 to 60 was determined by the cross-validation (CV) procedure of the program and the value with a minimum CV error was selected. The same set of 239,507 SNPs was used to perform a principal component analysis (PCA) as implemented in the SNPRelate (v1.26.0) program (Zheng et al., 2012).

The nucleotide diversity (π, the average number of nucleotide differences per site) and the nucleotide polymorphism (θ, the proportion of nucleotide sites that are expected to be polymorphic) of the collection of the 612 isolates and each population or group were calculated using the software Variscan (v2.0.6) (Hutter et al., 2006) with the NumNuc parameter being adjusted for each group for including at least 80% of isolates within the group and parameters ‘CompleteDeletion = 0, FixNum = 0, RunMode = 12, and WindowType = 0’ were selected.

### Gene Prediction and Gene Content Venn and PCA Analyses

In the study of Peter et al. (2018), a total of 7,796 ORFs were identified from the pan genome of *S. cerevisiae*, including 4,940 core ORFs and 2,856 variable ORFs. In this study, ORFs from the genomes of 368 isolates that were not employed in Peter et al. (2018) were predicted using Augustus (v2.5.5) (Stanke et al., 2004) with parameters mentioned above in genome annotation, then all predicted ORFs were aligned to the 7,796 ORFs using BLAST with the following parameters: gapopen 5, gapextend 5, penalty 5, reward 1, evalue 10, word_size 11, and no_greedy. A predicted ORF with an alignment identity of over 95% and an over 75% mapping length to one of the 7,796 ORFs identified in Peter et al. (2018) were regarded as a known ORF. Finally, 92 new ORFs were identified and a total of 7,888 ORFs were recognized as pan genome of *S. cerevisiae* in this study.

Then the assembled genome of each isolate employed in this study was executed BLAST search against the 7,888 ORFs with the parameters mentioned above in the novel ORFs prediction process. The presence of an ORF in an isolate was determined by the threshold of 95% alignment identity and 75% mapping length. To avoid the effects of rare ORFs and prediction bias, we removed the ORFs which were present in less than 1% of all the isolates compared. Finally, 6,999 ORFs were remained for Venn and PCA analyses based on the presence or absence of each of these ORFs in each strain compared. Venn diagram analysis was performed using the R package VennDiagram (Chen and Boutros, 2011). The package Python-TSNE^2^ was used to reduce the dimensionality of the gene contents of the isolates compared.

The functions of ORFs which are not present in the genome of S288c were estimated by annotations in the Pfam database (Finn et al., 2014) and online protein BLAST search^3^.

### Allele Distribution Analysis

From the 1,382,078 SNPs employed in phylogenomic analysis, the sites which were shared by less than 1% isolates were filtered out and 595,790 sites were remained. Then 10,000 SNPs were randomly selected from the remained sites by custom python script. Finally, we excluded the sites that exist in less than 80% isolates of any lineages, resulting in 7,348 sites for further analyses. The alleles were ordered by the groups and lineages, and then visualized using ggplot2 (Wickham, 2016). Group specific alleles were defined as the alleles that were shared by more than 90% isolates in one domesticated group but not shared by more than 90% isolates in the other domesticated group. BEDTools (Quinlan and Hall, 2010) was used to map group specific SNPs to genes annotated in *S. cerevisiae* S288c.

Gene Ontology (GO) enrichment analysis was implemented using Metascape (Zhou et al., 2019) and the GO terms with corrected *P* < 0.01 were considered as significantly enriched. Enrichment networks were then constructed based on functional similarities of the terms which were measured using an algorithm adopts Kappa statistics (Huang et al., 2009). The algorithm quantitatively measures the degree of the agreement how genes share the similar annotation terms, resulting in Kappa similarities ranging from 0 to 1. Enrichment networks were created by representing each enriched term as a node and connecting pairs of nodes with Kappa similarities above 0.3 (Zhou et al., 2019).

### Data Processing, Statistical Analyses, and Visualization

Primary data processing and conversion were performed using the Python project (v3.7.0)^4^. Standard statistical analyses were conducted in R project (v3.3.5)^5^ with custom scripts under available packages in the project. Figtree (v1.44)^6^ and iTOL (Letunic and Bork, 2016) were used for visualizing phylogenetic trees. R package ggplot2 (Wickham, 2016) was employed for visualizing other results.

## Results

### The Population Structure of *S. cerevisiae* Is Primarily Shaped by Ecology

**FIGURE 1:**
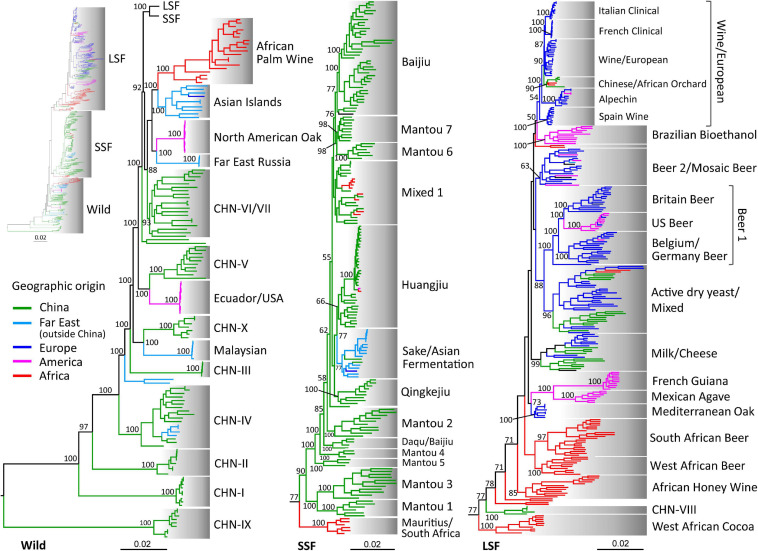
Phylogeny of wild and domesticated isolates of *S. cerevisiae* inferred from maximum likelihood analysis based on 1,382,078 genome-wide SNPs from 612 isolates with worldwide origins. The tree is rooted by lineage CHN-IX and the bootstrap support values to lineages and major clades above 50% are shown. Strain branches are colored according to geographic origins. LSF, liquid-state fermentation; SSF, solid-state fermentation. Scale bar, 0.02 substitutions per site.

In this study we sequenced 126 isolates, including 64 African strains from various indigenous fermented foods and forests in Ethiopia, Mauritius, Nigeria, and South Africa and 52 strains associated with Baijiu (Chinese liquor) fermentation and 10 strains associated with Huangjiu (rice wine) fermentation from China (Supplementary Table 1). A total of 486 previously sequenced strains representing all lineages that were recognized in previous studies (Gallone et al., 2016; Duan et al., 2018; Peter et al., 2018) were selected for an integrated phylogenomic analysis (Supplementary Table 1). In agreement with Duan et al. (2018), three major groups were resolved in the phylogenetic tree constructed from the whole genome SNPs, covering a total of 1,382,078 sites (Supplementary Figure 1). The wild and the domesticated populations were clearly separated. The domesticated strains were clustered into two major groups associated mainly with liquid- and solid-state fermentation, respectively (Supplementary Figure 1). The lineages are defined and named in this study based on a combined consideration of phylogenetic clustering, ecological and geographic origins, population structures and definitions and names used in previous studies. A total of 42 lineages are recognized in this study and 14 lineages are recognized in each of the Wild, the SSF, and the LSF groups. Distinct sub-lineages in the Wine/Europe and Beer 1 lineages are also recognized as in Peter et al. (2018) and Gallone et al. (2016) (Figure 1, Supplementary Table 1, and Supplementary Figures 1,2).

The wild group recognized in this study mainly consists of forest strains from China, other Far East countries (including Japan, Malaysia, and Russia), and North and South America (Figure 1 and Supplementary Figures 1,2). The CH-IX lineage from a primeval forest in Central China and the Taiwanese lineage from soil which were recognized as the oldest lineage of *S. cerevisiae* in Duan et al. (2018) and Peter et al. (2018), respectively, were clustered together (Figure 1 and Supplementary Table 1). Differing from Pontes et al. (2020) which combined the North America-Japan group (Almeida et al., 2015), the Far East Russia group (Peter et al., 2018) and the China VI–VII group (Duan et al., 2018) in one lineage (Clade 17), these groups are all resolved as distinct lineages in the Wild group this study (Figure 1 and Supplementary Figure 2). The Ecuador/United States lineage consisted of strains from trees, fruit, flowers, and insects was closely related with the CHN-V lineage from South China forests. The North American Oak lineage formed a sister clade of the Far East Russia lineage (Figure 1). We did not identify any new wild lineages or wild isolates belonging to basal wild lineages of *S. cerevisiae* from Africa. The Asian Islands lineage containing strains from trees, unspecified fermentation environment and palm wine in Indonesia, Pakistan, Philippines, and Sri Lanka (Peter et al., 2018) formed a sister clade of the African Palm Wine lineage containing strains from Burkina Faso, Djibouti, and Nigeria. These two lineages are mostly from spontaneous fermentation environments and closely related with the domesticated population (Figure 1 and Supplementary Figures 1,2).

The LSF group recognized in our previous study (Duan et al., 2018) is substantially expanded by additional strains from different continents. The European industrial strains for beer, bread and wine production compared in Gallone et al. (2016) and Peter et al. (2018) were included in this group. Interestingly, African strains clustered in this group were mostly located in basal lineages. The strains from honey wine collected in Ethiopia formed a distinct basal lineage. The strains associated with beer brewing in Africa form other two strongly supported basal lineages. One is formed by beer strains from South African and the other by beer strains from West African countries, including Chad, Ghana, Ivory Coast and Nigeria (Figure 1 and Supplementary Table 1). The African beer lineages were clustered together, but they are not statistically supported. Strains associated with cocoa fermentation in West Africa formed a root lineage of the LSF group. The strains from Mexican agave distillery and French Guiana form two closely related lineages. The Mediterranean oak (MO) lineage, which was regarded as the ancestor of European wine strains (Almeida et al., 2015), shows a close relationship to the two South American lineages with a moderate support. The strains in the Milk lineage recognized in Duan et al. (2018) and the French dairy lineage recognized in Legras et al. (2018) and Peter et al. (2018) merge in a distinct lineage (Figure 1). In agreement with Peter et al. (2018), the strains used in Brazilian bioethanol production form a distinct lineage with a weakly supported close relationship to the Wine/European lineage. Six sub-lineages are recognized in the Wine/European lineage (Figure 1 and Supplementary Table 1). The Spain wine, Alpechin, French clinical, and Italian clinical strains form well-supported sub-lineages, respectively. Four strains from Orchard in Northwest China and two strains from oak tree in South Africa form another sub-lineage.

As shown in Duan et al. (2018), the SSF group is mainly consisted of domesticated strains from China. The Sake/Asian Fermentation lineage recognized in Peter et al. (2018) is closely related with the Huangjiu (rice wine) lineage recognized in Duan et al. (2018). A nature strain from Ecuador, South America, and a soil strain from Algeria, Africa, formed a branch within the Huangjiu lineage. Three strains from cow milk and four strains from forest in South Africa and one strain from tree in Mauritius locate in a mixed lineage consisted of strains associated with various solid-state fermentation processes in China (Figure 1 and Supplementary Table 1). Five African strains from fruit trees and juice collected in Mauritius and South Africa formed a lineage basal to the SSF group, but with a moderate support.

After removing the SNPs with a minor allele frequency of < 0.05 and those in linkage-disequilibrium according to Gallone et al. (2016), a principle component analysis (PCA) was performed based on 239,507 sites. The result shows that the typical wild and domesticated strains are also clearly separated (Supplementary Figure 3). The most ancient lineages CHN-IX, CHN-I, and CHN-II are clearly separated from the other wild strains. The LSF and SSF groups are also clearly separated. The Mauritius/South Africa lineage which is located basal to the SSF group in the phylogenetic tree (Figure 1) is located between the wild and SSF groups. Some strains in the West African Cocoa and CHN-VIII lineages, which are resolved as the basal lineages of the LSF group, are located between the wild and LSF groups (Supplementary Figure 3).

### Variations in Sequence Diversity and Heterozygosity

**FIGURE 2:**
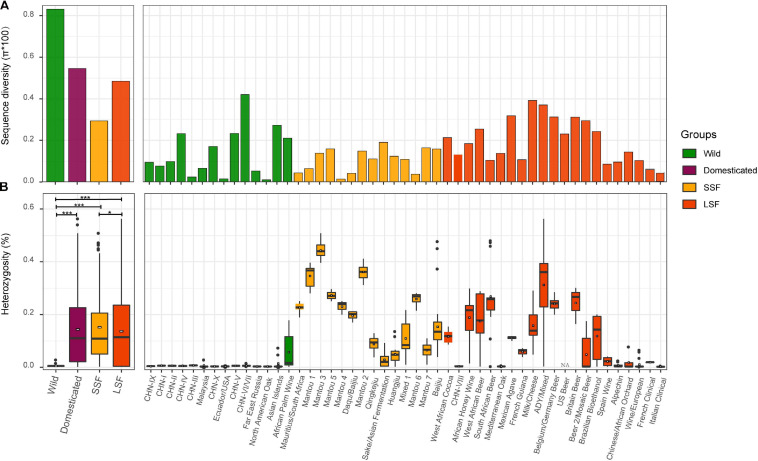
Sequence diversity **(A)** and heterozygosity **(B)** of different populations, groups and lineages of *S. cerevisiae*. Sequence diversity **(A)** is calculated from genome-wide SNPs, and heterozygosity **(B)** is expressed as the ratio of heterozygous SNPs to the consensus genome size of each isolate. LSF, liquid-state fermentation; SSF, solid-state fermentation. *, *P* < 0.05; ***, *P* < 0.001.

Though the domesticated population is substantially expanded compared to our previous study Duan et al. (2018), especially the LSF group, the sequence diversity of the wild population (π = 8.08 × 10^–3^) is still higher (1.48-fold) than that of the domesticated population (π = 5.46 × 10^–3^) (Figure 2A). Within the domesticated population, the sequence diversity of the LSF group (π = 4.85 × 10^–3^) is higher (1.65-fold) than that of the SSF group (π = 2.94 × 10^–3^). At the lineage level, the wild lineage CHN-VI/VII (π = 4.21 × 10^–3^) exhibited the highest genetic diversity, followed by the domesticated lineage milk/cheese (π = 3.92 × 10^–3^) (Figure 2B).

As shown in our previous study (Duan et al., 2018), the wild and domesticated strains exhibited a hallmark difference in heterozygosity (Figure 2C). The wild isolates are generally homozygous with an average heterozygous site ratio of 0.005%, being significantly lower than that of the domesticated isolates (0.143%, *P* < 0.001). The average heterozygosity of the LSF group (0.137%) is slightly lower than that (0.152%) of the SSF group (*P* = 0.039). Different lineages in both groups exhibited remarkably different degree of heterozygosity (Figure 2D). Isolates with exceptionally high heterozygosity are mostly from Africa. For example, the two isolates exhibiting the highest heterozygosity (0.54% and 0.56% heterozygous site ratio, respectively) are Ethiopian honey wine strains located in the Active dry yeast/Mixed lineage in the LSF group (Figure 2D and Supplementary Table 1). However, on average, the three lineages formed by strains associated with Mantou (steamed bread) fermentation in China possessed the highest heterozygosity (0.346–0.443%) among the domesticated lineages (Figure 2B). It is worth noting that though the forest strains in the CHN-VIII and the Mediterranean Oak lineages are clustered in the LSF group in the phylogenetic tree (Figure 1), they exhibited as low heterozygosity as the wild strains (Figure 2D). The strains in the Wine/European lineage exhibited relatively low heterozygosity compared to other domesticated lineages. It is consistent with the observation that the Wine/European lineage contains the highest proportion of homozygous isolates among domesticated lineages of *S. cerevisiae* (Peter et al., 2018).

### Gene Content Variations Recapitulate Population Differentiation

**FIGURE 3:**
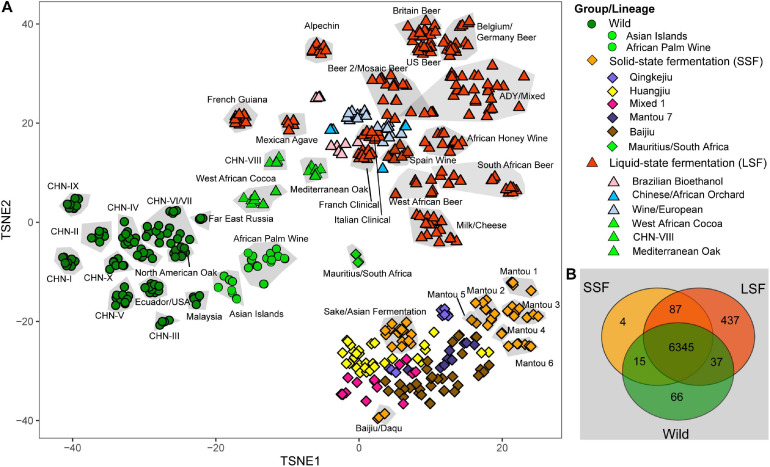
Gene content variations in the genomes of *S. cerevisiae* isolates. **(A)** Principal component analysis (PCA) based on the presence or absence of each gene in each isolate. When the isolates belonging to the same lineage form a distinct clustered, they are included in the same gray block. The lineages with isolates that are not clustered together are marked using different colors. **(B)** Venn diagram based on gene contents. The numbers of genes which are shared by different groups or occur exclusively in single groups are shown.

A total of 7,888 ORFs, including 92 ORFs that were not identified by Peter et al. (2018), were identified in the isolates compared in this study. After excluding the ORFs that exist in only single strains or shared by less than 1% of the strains compared, we identified 6,999 ORFs from the 612 isolates for further analyses. PCA analysis based on the presence or absence of each of the ORFs in each strain showed that the Wild and the two domesticated LSF and SSF groups were also clearly separated (Figure 3A). The strains from the same lineages were also generally clustered together, except a few domesticated lineages, including three lineages in the LSF group and five lineages in the SSF group (Figure 3A). Six lineages, including the Asian Island, African Palm Wine, Mauritius/South Africa, West African Cocoa, CHN-VIII and MO, were located between the wild and domesticated groups (Figure 3A).

We then tried to find genes that exist only in specific groups or lineages by Venn analysis. A total of 6,345 genes were shared by the three main groups and 69, 4 and 436 genes were found only in the Wild, SSF and LSF groups, respectively (Figure 3B). Genes in the regions A, B, and C, which were obtained by HGT and first found in the wine yeast strain EC1118 (Novo et al., 2009), were found to be LSF group specific genes. The genes in the regions A, B, and C were completely absent in the wild strains compared in this study (Supplementary Figure 4). They are also absent in the SSF group, except in two strains (AHQ and BEF) in the Sake/Asian Fermentation lineage which have five genes of region B and three genes of region C, respectively (Supplementary Figure 4). Genes in Region A were detected in only four strains of the Beer2/Mosaic Beer lineage. Region B genes were detected in 45% isolates and 90% lineages of the LSF group. Region C genes distributed in 26% strains and 40% lineages of the LSF group (Supplementary Figure 4).

From the group specific genes, we identified 347 lineage specific genes (existing in only single lineages) from 15 lineages (Supplementary Table 2). Among these genes, 261 were introgressed from *S. paradoxus* (Supplementary Table 2). Notably, the Alpechin (waste from olive oil production) lineage has as many as 214 unique genes and 203 (94.9%) of them were introgressed from *S. paradoxus* (Supplementary Table 2). This result is in agreement with the finding that the *S. cerevisiae* lineage associated with processed olives results from a hybridization between *S. cerevisiae* and *S. paradoxus* (Pontes et al., 2019). The majority of the lineage specific genes in the French Guiana (92.9%) and the Mexican Agave (57.1%) lineages also originate from *S. paradoxus* (Supplementary Table 2), implying a similar hybridization history of the two yeast lineages in South America.

**FIGURE 4:**
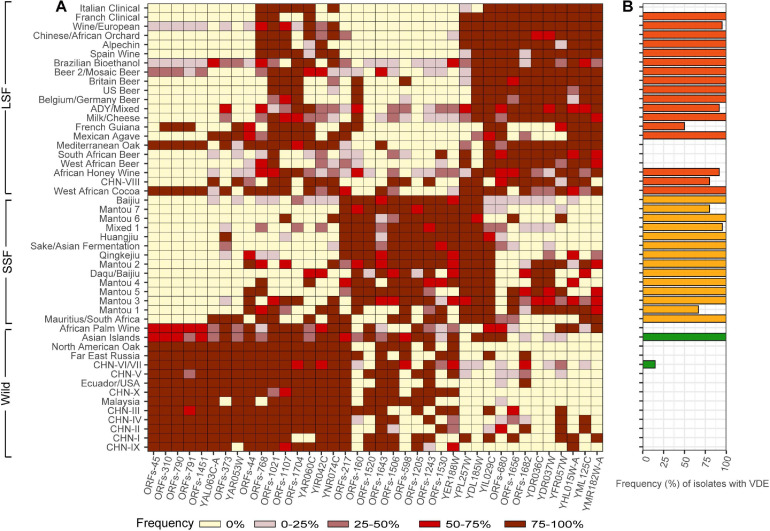
Distribution of selected genes in different populations, groups and lineages of *S. cerevisiae*. **(A)** Frequencies of isolates in different lineages harboring the genes listed at the bottom. These genes are shared by the majority (>90%) of the isolates in one group but occur in limited isolates (<10%) in another group. Frequency ranges are depicted using different colors as shown at the bottom. **(B)** Frequencies of isolates harboring the homing endonuclease VDE in gene *VMA1* in different lineages in the wild (dark green), liquid- (red) and solid-state fermentation (orange) groups.

The group specific genes identified by the Venn analysis occurs in only limited lineages or strains of the group and we did not identify group specific genes that were shared by more than 50% of the strains in each group. We then tried to find genes that are shared by the majority (>90%) of the strains in one group but occur in limited strains (<10%) in another group. We finally identified 38 genes which are shared by the majority of the strains in the same groups (Figure 4A). These genes all fall in the set of variable OFRs defined in Peter et al. (2018) except YDL185W (*VMA1*). These genes likely contribute to the adaptation of different groups to the wild or different fermentation environments, but the functions of many of these genes are still unclear (Supplementary Table 3).

The gene YDL185W (*VMA1*) is worth noting. This gene harbors an intein VDE (*VMA1*-derived endonuclease) which is a nuclear homing endonuclease that has been found from several yeast species (Koufopanou et al., 2002). Two versions of *VMA1*, with and without VDE, respectively, distribute in the *S. cerevisiae* strains compared. Up to 90.6% strains in the wild population do not harbor VDE (Figure 4B). Specifically, the strains in the basal wild lineages completely lack VDE. Only three strains in lineage CHN-VI/VII mostly from secondary forests and one Mosaic isolate (YN3 from soil) and the ten strains in the Asian Islands lineage closely related with the domesticated populations have VDE. On the other hand, VDE commonly exists in domesticated strains and 97.2% SSF and 79.4% LSF isolates have VDE. In the majority of the domesticated lineages recognized, 100% strains tested contain VDE. Only three domesticated lineages (South African Beer, West African Beer and Italian Clinical) do not have VDE (Figure 4B).

### Group- and Lineage-Specific Sequence Divergence of Core Genes

In order to investigate sequence divergence of core genes of the species, we performed SNP distribution and enrichment analysis. We randomly selected 10,000 SNPs from the total SNPs identified and then excluded the sites that exist in less than 80% isolates of any lineages, resulting in 7,348 sites for further analyses. We found evident group and lineage specific allele distribution patterns (Supplementary Figure 5). The alleles shared by the strains in the LSF and SSF groups were clearly different. In addition, the majority of the lineages possessed lineage specific alleles. Notably, all wild lineages except lineage CHN-VI/VII have evident lineage specific alleles and the basal lineages CHN-IX, CHN-I and CHN-II have more lineage specific alleles than others. In the domesticated population, the LSF group possessed more evident lineage specific alleles than the SSF group. Many lineage-specific alleles in the domesticated population were heterozygous (Supplementary Figure 5).

**FIGURE 5:**
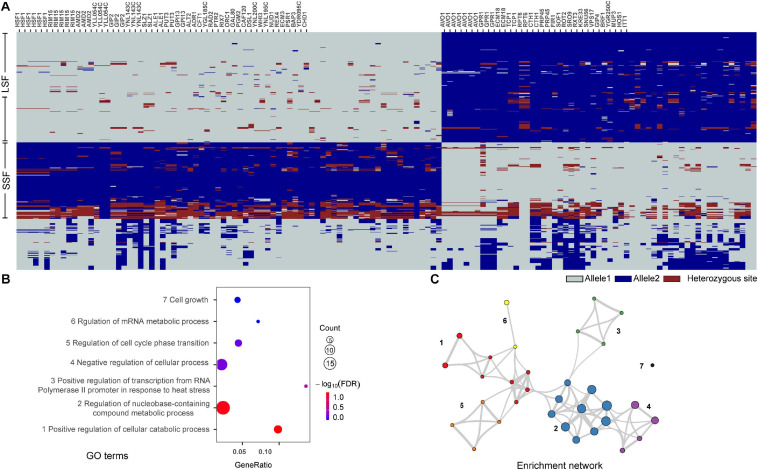
Functions of genes with group specific allele distribution patterns in different domesticated groups of *S. cerevisiae*. **(A)** Distribution patterns of selected SNPs with alleles that were shared by more than 90% isolates in one domesticated group but not shared by more than 90% isolates in the other group. The genes with known functions harboring the SNPs are listed at the top. **(B)** GO terms enriched with the genes with group specific allele distribution patterns. **(C)** Enrichment networks show the intra- and intercluster functional similarities of the enriched GO terms shown in **(B)**. Enrichment networks are created by representing each enriched term as a node and connecting pairs of nodes with Kappa similarities above 0.3. The sizes of the nodes are proportional to the numbers of input genes fall into the terms.

To figure out genes harboring group specific alleles, we selected SNPs with alleles that were shared by more than 90% isolates in one domesticated group but not shared by more than 90% isolates in the other domesticated group for enrichment analysis. A total of 134 sites were identified and 88 of them were mapped to 54 known genes annotated in *S. cerevisiae* S288c (Figure 5 and Supplementary Table 4). The alleles containing these SNP sites showed clearly different distribution patterns between the wild and the domesticated populations and between the two domesticated groups (Figure 5A and Supplementary Table 4). Gene Ontology (GO) enrichment analysis using the Metascape tool (Zhou et al., 2019) showed that the 54 genes were enriched in seven terms, including positive regulation of cellular catabolic process, regulation of nucleobase-containing compound metabolic process, positive regulation of transcription from RNA polymerase II promoter in response to heat stress, negative regulation of cellular process, regulation of cell cycle phase transition, regulation of mRNA metabolic process, and cell growth (Figure 5B). Interestingly, six of the seven GO terms formed an enrichment network resulted from the Metascape analysis based on quantitative measurements of the functional similarities of the GO terms (Figure 5C) (Zhou et al., 2019). The result suggests that the genes with group specific alleles are generally similar or correlated in function.

**FIGURE 6:**
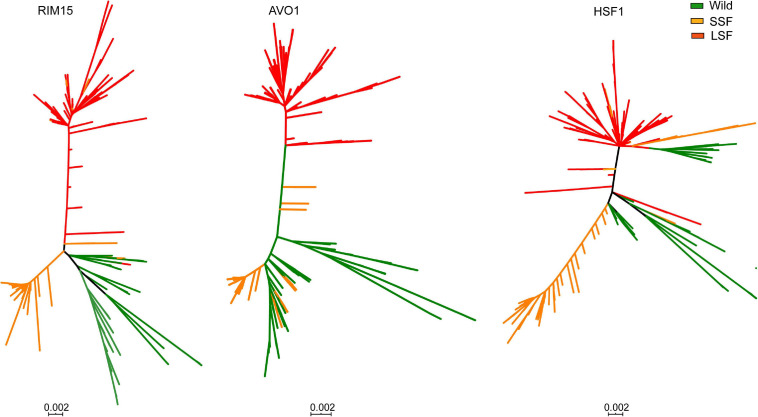
Unrooted phylogenetic trees of 612 worldwide isolates of *S. cerevisiae* constructed from the sequences of genes *AVO1*, *HSF1*, and *RIM15*, respectively. Isolate branches are colored according to the positions of the isolates clustered in the wild, liquid-state (LSF) and solid-state fermentation (SSF) groups in the tree constructed from the whole genome SNPs as shown in Supplementary Figure 1.

Among the 54 genes identified, genes *HSF1*, *RIM15*, and *AVO1* harboring the highest number (5–7) of group specific SNPs each in the LSF and SSF groups (Figure 5A). Phylogenetic analyses based on the sequences of these genes showed that the topologies of the single gene trees are similar with that of the tree constructed from the whole genome SNPs (Figure 6). The three main groups recognized based on the whole genome SNP analysis were largely resolved in the single gene trees. The result suggests that the sequence divergence of these genes correlates well with the divergence of the domesticated groups and likely contribute to the ecological adaption of the groups.

## Discussion

The present phylogenomic analysis on a set of worldwide *S. cerevisiae* strains representing the maximum genetic diversity of the species documented so far confirms our previous conclusion that the divergence and population structure of the species are primarily shaped by ecology (Duan et al., 2018). Wild and domesticated populations are clearly separated, supporting our hypothesis that the domesticated population probably originated from a single domestication event (Duan et al., 2018). The separation of the two main groups generally associated with solid-state and liquid-state fermentation, respectively, in the domesticated population remains clear after more African strains are included. The LSF group includes native lineages from Africa, America, Asia, and Europe (Figure 1 and Supplementary Figures 1,2). Though the SSF group includes strains mainly from Far East Asian countries, a few African strains are located in this group. Solid-state fermentation is also employed in fermented food production in Africa, for examples, teff (*Eragrostis tef*) dough and false banana (*Ensete ventricosum*) starchy pulp (bulla) fermentation (Ashenafi, 2008; Koricha et al., 2020). It is reasonable to expect to find more isolates belonging to the SSF group from Africa.

Lineages within the wild and domesticated population are shaped by both ecology and geography. Geography seems to play a main role in the wild population. Wild strains from forests in North and South America are located in the wild population together with forest strains from Far East Asia, but they form distinct lineages. Strains from Far East countries other than China also form separate lineages from Chinese strains. Ecology seems to be the main driving force for the divergence of domesticated strains. Within each of the two domesticated groups, strains used for different food and beverage fermentation usually formed distinct lineages, while those associated with the same fermentation environments usually cluster together, regardless of their geographic origins. For example, strains from fermented milk products in Asia and Europe are located together in a single lineage. Geography may also play a role in the diversification of domesticated strains, especially for the strains associated with Mantou fermentation (Figure 1) and beer brewing (Gallone et al., 2016). The strains associated with Mantou fermentation from different regions or provinces of China form several distinct lineages (Mantou 1 to Mantou 7). The beer strains from South Africa form a lineage separate from that formed by the beer strains from west African countries (Figure 1). However, the role of ecological factor for the differentiation of the African beer strains cannot be excluded, because the raw materials and fermentation processes may be different in different African countries.

The types of substrates involved in the liquid- (grape juice, wort, milk, etc.) and solid-sate (dough, sorghum grain, barley grain, cooked rice, etc.) fermentations are diversified and should contribute to the diversification of the yeast strains involved within each of the two groups. The clear separation of the LSF and SSF yeast groups suggests that there should be a common selective pressure for the divergence of the two groups. The main difference between the two types of fermentation is the water contents of the fermentation substrates. The water contents are usually 80–90% and 40–60% in the liquid- and solid-state fermentation, respectively. The water contents in the substrates of Huangjiu and Sake (cooked rice in semi-solid state) is usually 70–80%, but the starter (Jiuqu) of Huangjiu, which provides yeasts and other microbes for the fermentation process, is prepared through solid-state fermentation of wheat grain with a water content of about 40–50%. The Sake yeast, which is usually inoculated in pure culture at the beginning of fermentation, probably originated from Huangjiu yeast as shown in Duan et al. (2018) and the present study (Figure 1).

Within each of the Wild, LSF and SSF groups recognized in the phylogenetic tree based on genome SNPs (Figure 1), there are lineages that are not from typical environments of the groups. These lineages include the Asian Island lineage with strains from palm wine and the African Palm Wine lineage in the wild group; the Mauritius/South Africa lineage with strains from fruit trees and juice in the SSF group; and the CHN-VIII lineage with strains from fruit and secondary forests, the West African Cocoa lineage, and the MO lineage in the LSF group. In the PCA analysis based on the gene content, they are located between the wild and domesticated groups (Figure 3A), implying that these lineages probably represent transitional forms between the wild and domesticated populations of *S. cerevisiae*. Interestingly, Pontes et al. (2020) detected domestication signatures from one tree bark and one fruit strain of the CHN-VIII lineage. It is also possible that the strains in the MO lineage, which locates in the middle of the LSF group, are escapees from a certain liquid-state fermentation environment.

We have proposed a China/Far East Asia origin hypothesis of the domesticated population of *S. cerevisiae* based on our previous study with limited African strains (Duan et al., 2018). However, the present study shows that African strains are located in basal lineages of the LSF and SSF groups, implying an alternative African origin hypothesis of the domesticated population of *S. cerevisiae*, as proposed by Fay and Benavides (2005). To resolve the problem, more wild and domesticated African strains need to be sampled and sequenced. Africa is one of the origin centers of domesticated plants in the world and is rich in diversified fermented foods and beverages, in which *S. cerevisiae* is usually among the essential functional microorganisms (Koricha et al., 2020). The present study shows that *S. cerevisiae* strains from different fermented food samples in different African countries form distinct lineages, suggesting a potentially high genetic diversity of the domesticated population of *S. cerevisiae* in Africa.

The distribution and diversity of wild population of *S. cerevisiae* in Africa remain largely unknown. Though *S. cerevisiae* strains from forests in different African countries have been isolated, but they are not located in the wild population of the species. Four strains (SAN30 to SAN33) isolated from a forest in South Africa are located in a mixed lineage together with some strains from Daqu (starter of Baijiu) in the SSF group. However, their high degree of heterozygosity suggests that they are probably recent escapees or contaminants from fermented environment. It is still unclear whether wild strains belonging to basal wild lineages exist in Africa or not. Survey of wild *S. cerevisiae* in primeval forests in Africa will certainly be helpful to determine the origin place of the yeast in the world.

Differing from our single domestication event hypothesis, the multiple independent domestication events hypothesis (Liti et al., 2009; Peter et al., 2018) predicts that different domesticated lineages in different continents will have separate local wild ancestors. The wild ancestor (the MO lineage) of the Wine lineage was specified in Almeida et al. (2015). However, the close relationship between the Wine and the MO lineages as shown in Almeida et al. (2015) is not supported in the present study and in Pontes et al. (2020). When more isolates with more diversified origins were added to the phylogenetic tree, the MO lineage was separated from the Wine lineage and clustered together with two South American lineages Mexican Agave and French Guiana (Figure 1 and Supplementary Figures 1,2). The close relationship of the MO lineage with the South American lineages was also shown in Pontes et al. (2020). Multiple domesticated and wild lineages have been identified from each of the Asian and American continents; however, close relationships between the domesticated and wild lineages within each of the two continents are not observed. In contrast, as discussed above, the wild and domesticated lineages are respectively located in the same groups, regardless of their geographic origins (Figure 1 and Supplementary Figures 1,2). This result is apparently consistent with the single domestication event scenario. Further systematic investigation of indigenous wild and domesticated *S. cerevisiae* in Africa will also be of great value for testing the origin hypotheses of the domesticated lineages of *S. cerevisiae*.

Previous studies have revealed extensive genomic changes associated with ecological adaptation of domesticated *S. cerevisiae*, including heterozygosity, gene contraction or expansion, SNP accumulation and foreign gene acquisition through HGT or introgression (Magwene et al., 2011; Gallone et al., 2016; Steenwyk and Rokas, 2017; Duan et al., 2018; Peter et al., 2018; Pontes et al., 2020). However, hallmark genomic differences between the SSF and LSF groups have not been shown, though they are phylogenetically separated. In this study, we found more genomic variations between the wild and domesticated populations and between the domesticated SSF and LSF groups. We identified group and lineage specific genes which are likely associated with ecological adaption of the yeast (Figure 4 and Supplementary Tables 2,3). The specific roles or contribution of these genes to the adaption of different groups and lineages remain to be addressed, for the functions of the majority of these genes are largely unknown (Supplementary Table 3).

The clearly different distribution pattern of the homing intein VDE between the wild and domesticated populations of *S. cerevisiae* is interesting. The VDE coding gene is one of the homing endonuclease genes (HEGs) and appears to be specifically adapted for horizontal transmission (Koufopanou et al., 2002; Koufopanou and Burt, 2005). One possible explanation to the dominance of VDE in the domesticated but absent in the wild lineages of *S. cerevisiae* observed in this study is that VDE may have invaded into the initial domesticated ancestor formed by the single domestication event as proposed by Duan et al. (2018). Another possible explanation is that the invasion of VDE perhaps occurred recently in a fermentation environment and then spread among domesticated strains of *S. cerevisiae* through human assisted admixtures. Okuda et al. (2003) previously identified two subfamilies of VDE from nine of the 10 *S. cerevisiae* strains compared. The more extensive genomic resources available today for *S. cerevisiae* should allow a more thorough investigation of the intraspecific diversity of VDE in *S. cerevisiae*. We plan to carry out such studies in the future.

VDE and other intein-encoded HEGs are usually considered to be nonessential genes with no known function and susceptible to degeneration if they become fixed in a population (Koufopanou et al., 2002; Koufopanou and Burt, 2005). Little evidence for positive selection for intein-encoded HEGs by the host organism has been shown (Posey et al., 2004; Naor et al., 2016). However, a previous study shows that VDE is involved in the regulation of the high affinity glutathione transporter gene *GSH11* in *S. cerevisiae* (Miyake et al., 2003). The expression of *GSH11* is enhanced by rapamycin, an inhibitor of the TOR (target of rapamycin) signaling pathway, in a VDE-dependent manner under conditions of sulfur starvation (Miyake et al., 2003). Whether VDE is functional or plays a role in the adaptation of domesticated strains of *S. cerevisiae* as implied in this study remains to be revealed.

The evident group and lineage specific distribution of alleles of core genes of *S. cerevisiae* suggests that mutation or accumulation of SNPs plays an important role in the adaptation of different group or lineages to natural and specific fermentation environments (Figure 5 and Supplementary Figure 5). The genes with group specific allele distribution patterns are enriched in fundamental metabolism processes which are functionally similar or closely related (Figure 5). The clear difference in allele distribution of core genes in the domesticated LSF and SSF groups suggests that accumulation of SNPs not only play an important role in genome evolution of wild isolates as shown in Peter et al. (2018), but also in genome evolution and ecological adaptation of domesticated isolates. The genes *AVO1* and *HSF1* enriched with the highest number of group specific SNPs are involved in TOR signaling system. *AVO1* is an essential gene and involve in the regulation of cell growth and TOR signaling as a part of TORC2 complex. *HSF1* encodes a heat shock transcription factor which activates multiple genes in response to highly diverse stresses and negatively regulates TOR signaling pathway. Interestingly, the homing intein VDE with a group specific distribution pattern is also related with TOR signaling system as discussed above. In addition, the translocation of VDE is stimulated and stabilized by nitrogen starvation and inactivation of the TOR regulatory system as shown in Nagai et al. (2003). TOR signaling is an evolutionary conserved pathway that controls multiple cellular processes upon various intracellular and extracellular stimuli (Takahara and Maeda, 2013; Tatebe and Shiozaki, 2017). The present study suggests a possible role of TOR signaling pathway in the adaptation of wild and domesticated yeast strains to different natural and fermentation environments, through fixed mutations in genes involved in the pathway.

## Data Availability Statement

This datasets used in this study be found here: https://www.ncbi.nlm.nih.gov/bioproject/PRJNA680387.

## Author Contributions

F-YB and D-YH conceived and designed the project. F-YB, Q-MW, P-JH, KR, AK, and D-YH performed sampling and yeast isolation and identification. D-YH and J-YS isolated DNA for genome sequencing. D-YH, KL, LS, and S-FD performed the bioinformatics analyses. F-YB and D-YH analyzed the data and wrote the manuscript. All authors contributed to the article and approved the submitted version.

## Conflict of Interest

The authors declare that the research was conducted in the absence of any commercial or financial relationships that could be construed as a potential conflict of interest.

## Footnotes

1 https://sourceforge.net/projects/picard/

2 https://pypi.org/project/tsne/

3 https://blast.ncbi.nlm.nih.gov/Blast.cgi

4 https://www.python.org/

5 https://ww-w.rproject.org/

6 http://tree.bio.ed.ac.uk/software/figtree
