# Supplementary figures and images for: Adaptive Gene Content and Allele Distribution Variations in the Wild and Domesticated Populations of Saccharomyces cerevisiae
Source: Front Microbiol. 2021 Feb 17;12:631250. doi: 10.3389/fmicb.2021.631250 (PMC7925643; doi:10.3389/fmicb.2021.631250)

Figure S1

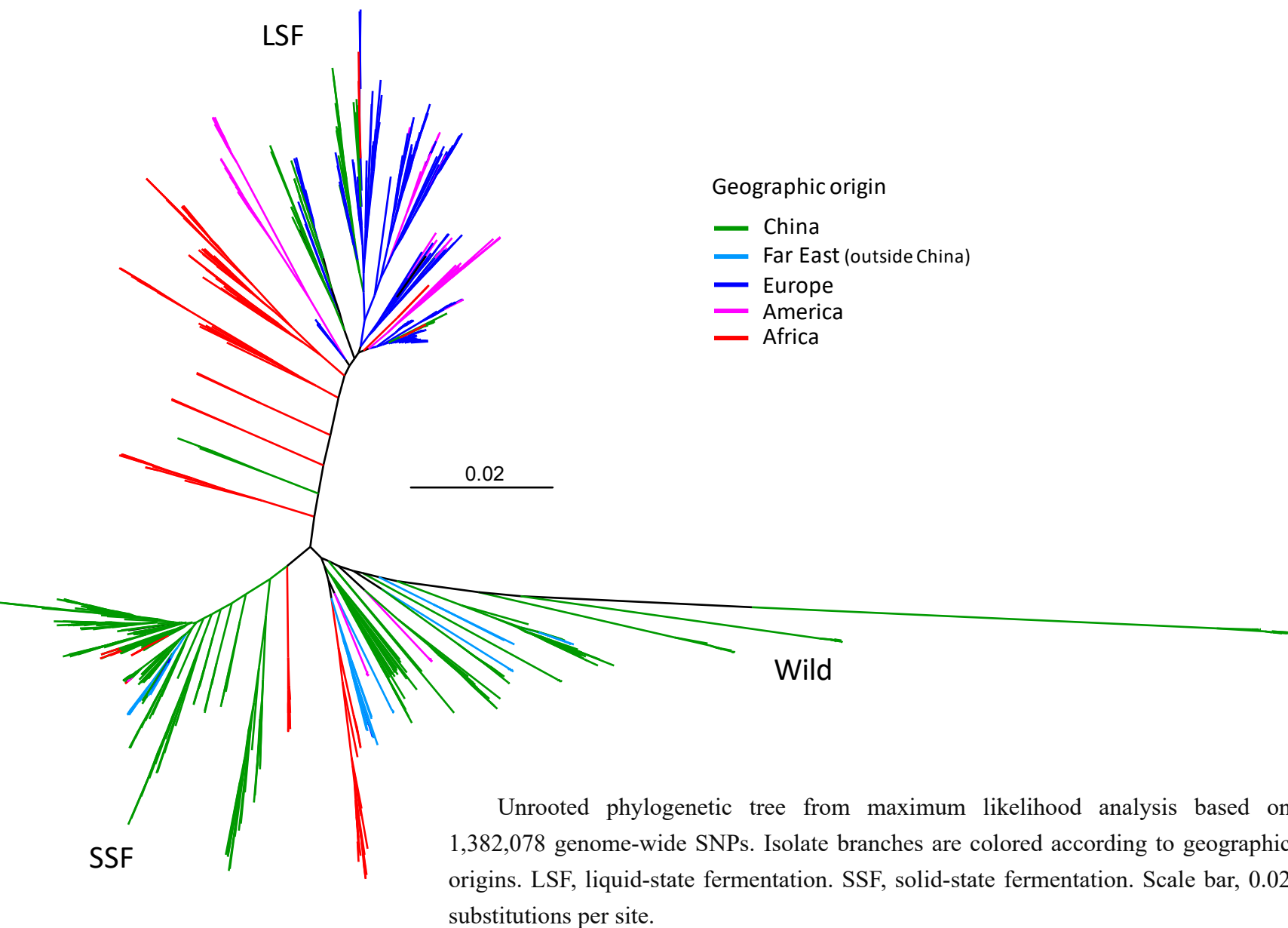

Supplement: Supplementary file 5 [file Image_1.pdf]
