# Supplementary material for: Adaptive Gene Content and Allele Distribution Variations in the Wild and Domesticated Populations of Saccharomyces cerevisiae
Source: Front Microbiol. 2021 Feb 17;12:631250. doi: 10.3389/fmicb.2021.631250 (PMC7925643; doi:10.3389/fmicb.2021.631250)
Supplement: Supplementary file 6 [file Image_2.pdf]

Figure S2

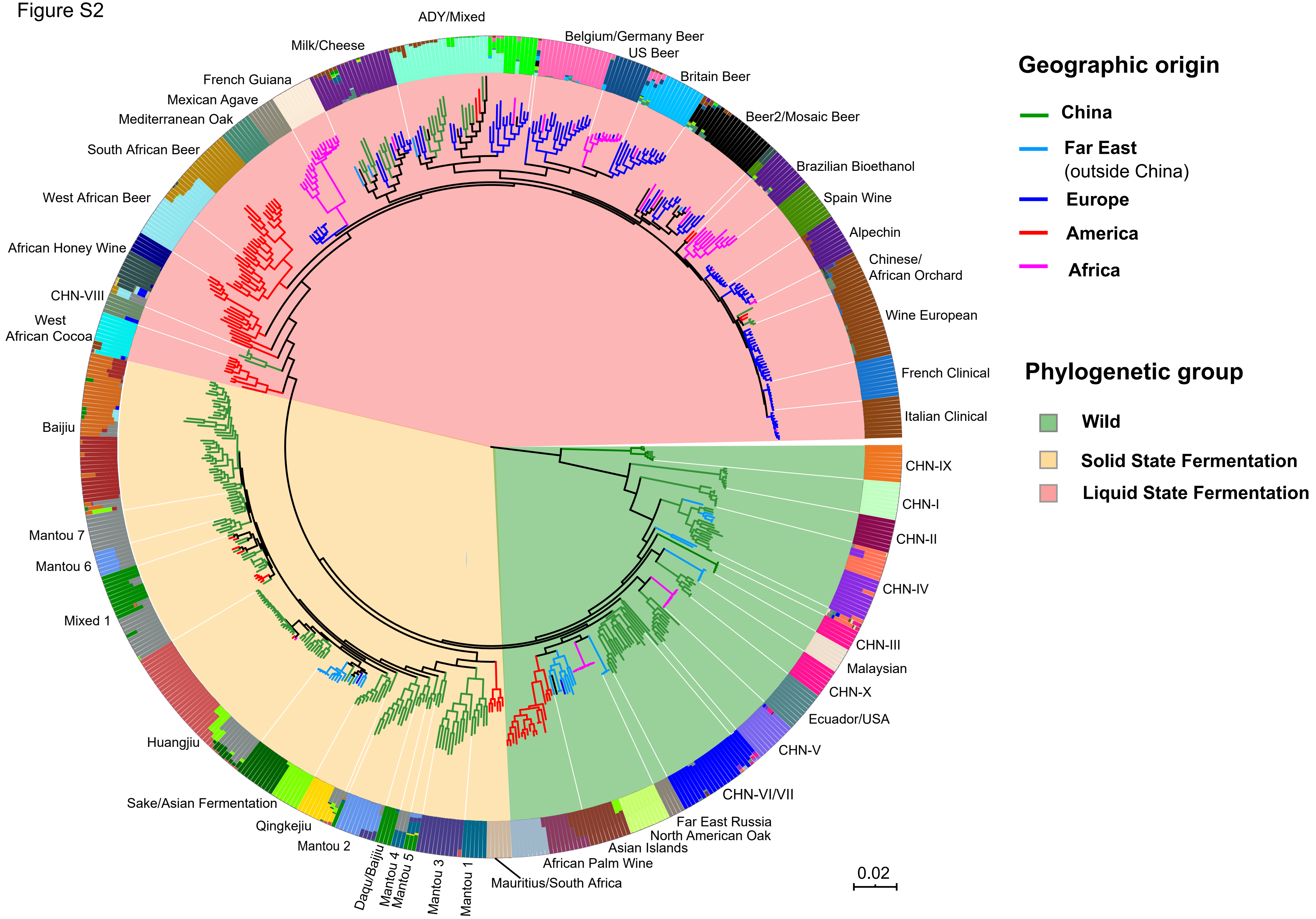

Phylogeny and population structure of 612 wild and domesticated isolates of S. cerevisiae with worldwide origins. The phylogenetic tree is constructed from maximum likelihood analysis of 1,382,078 genome-wide SNPs and rooted by lineage CHN-IX. Isolate branches are colored according to geographic origins and groups are marked by different background colors. The population structure shown in the outer ring is inferred using the ADMIXTURE program based on 239,507 biallelic SNPs when K (the number of populations assumed) is set to 47 as determined by the minimum cross-validation error check. Each strain is represented by a single vertical line broken into 47 colored segments, with lengths proportional to each of the 47 inferred clusters.
