# Supplementary material for: Adaptive Gene Content and Allele Distribution Variations in the Wild and Domesticated Populations of Saccharomyces cerevisiae
Source: Front Microbiol. 2021 Feb 17;12:631250. doi: 10.3389/fmicb.2021.631250 (PMC7925643; doi:10.3389/fmicb.2021.631250)
Supplement: Supplementary file 7 [file Image_3.pdf]

Figure S3

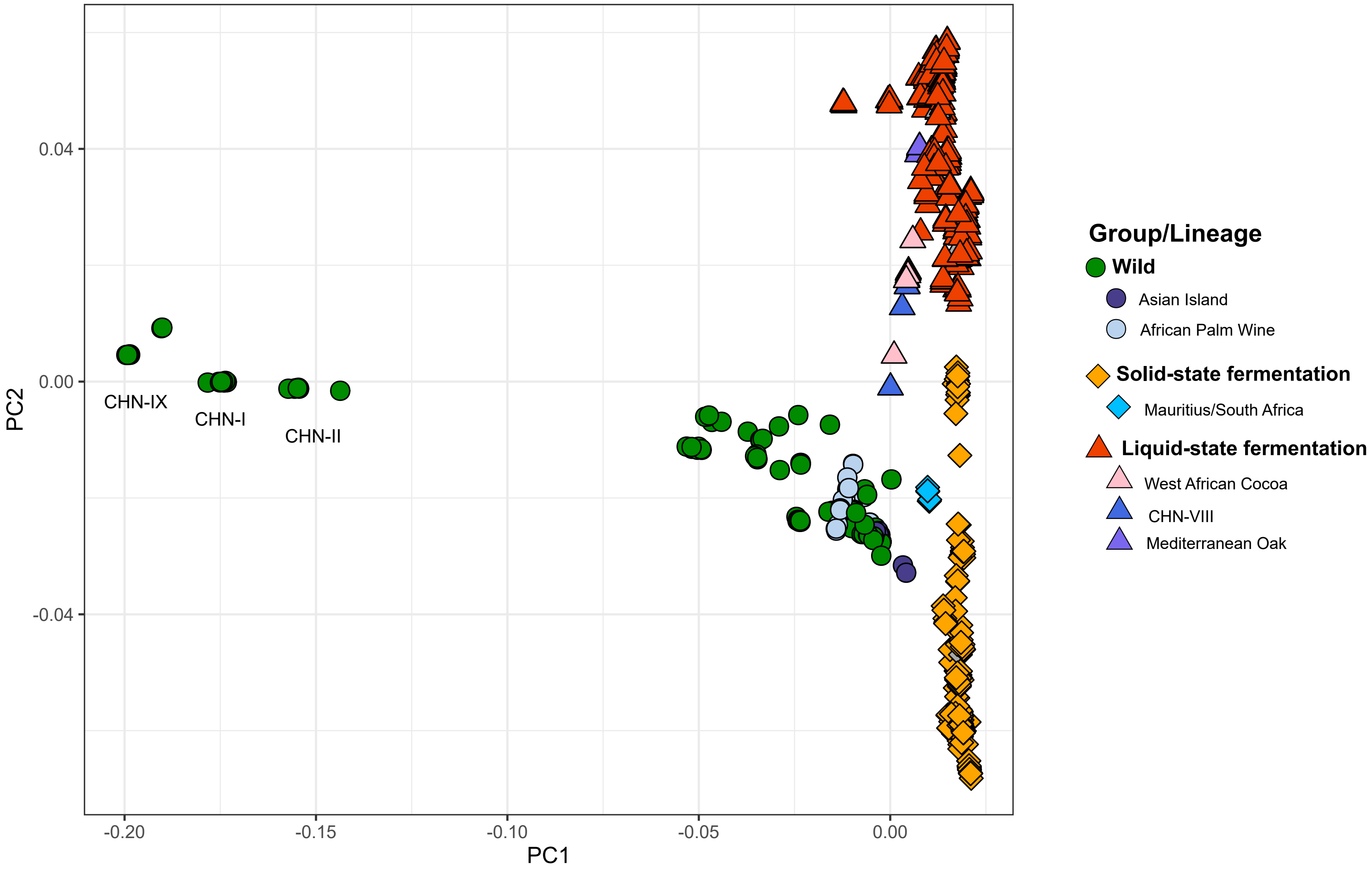

Principal component analysis (PCA) of genome wide SNPs from 612 wild and domesticated isolates of *S. cerevisiae* with worldwide origins
