# Supplementary material for: Adaptive Gene Content and Allele Distribution Variations in the Wild and Domesticated Populations of Saccharomyces cerevisiae
Source: Front Microbiol. 2021 Feb 17;12:631250. doi: 10.3389/fmicb.2021.631250 (PMC7925643; doi:10.3389/fmicb.2021.631250)
Supplement: Supplementary file 8 [file Image_4.pdf]

Figure S4

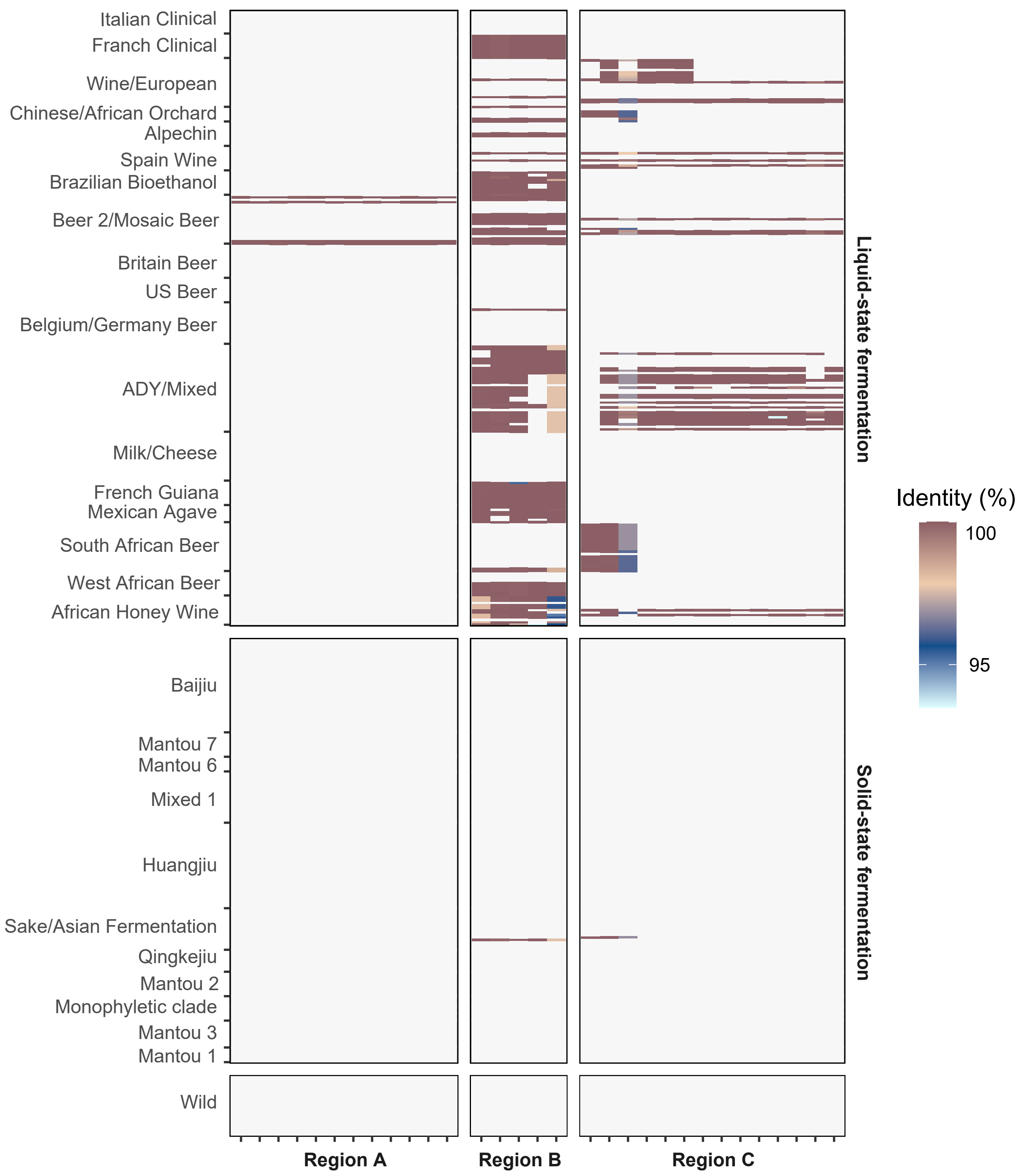

Distribution of genes in Regions A, B, and C in different groups and lineages of S. cerevisiae. The wild group are collapsed because the genes are absent in all the wild isolates compared. The sequence identity of each gene represented by a fragment at the bottom to the corresponding gene in each isolate is shown according to the scale on the right
