# Supplementary material for: Adaptive Gene Content and Allele Distribution Variations in the Wild and Domesticated Populations of Saccharomyces cerevisiae
Source: Front Microbiol. 2021 Feb 17;12:631250. doi: 10.3389/fmicb.2021.631250 (PMC7925643; doi:10.3389/fmicb.2021.631250)
Supplement: Supplementary file 9 [file Image_5.pdf]

Figure S5

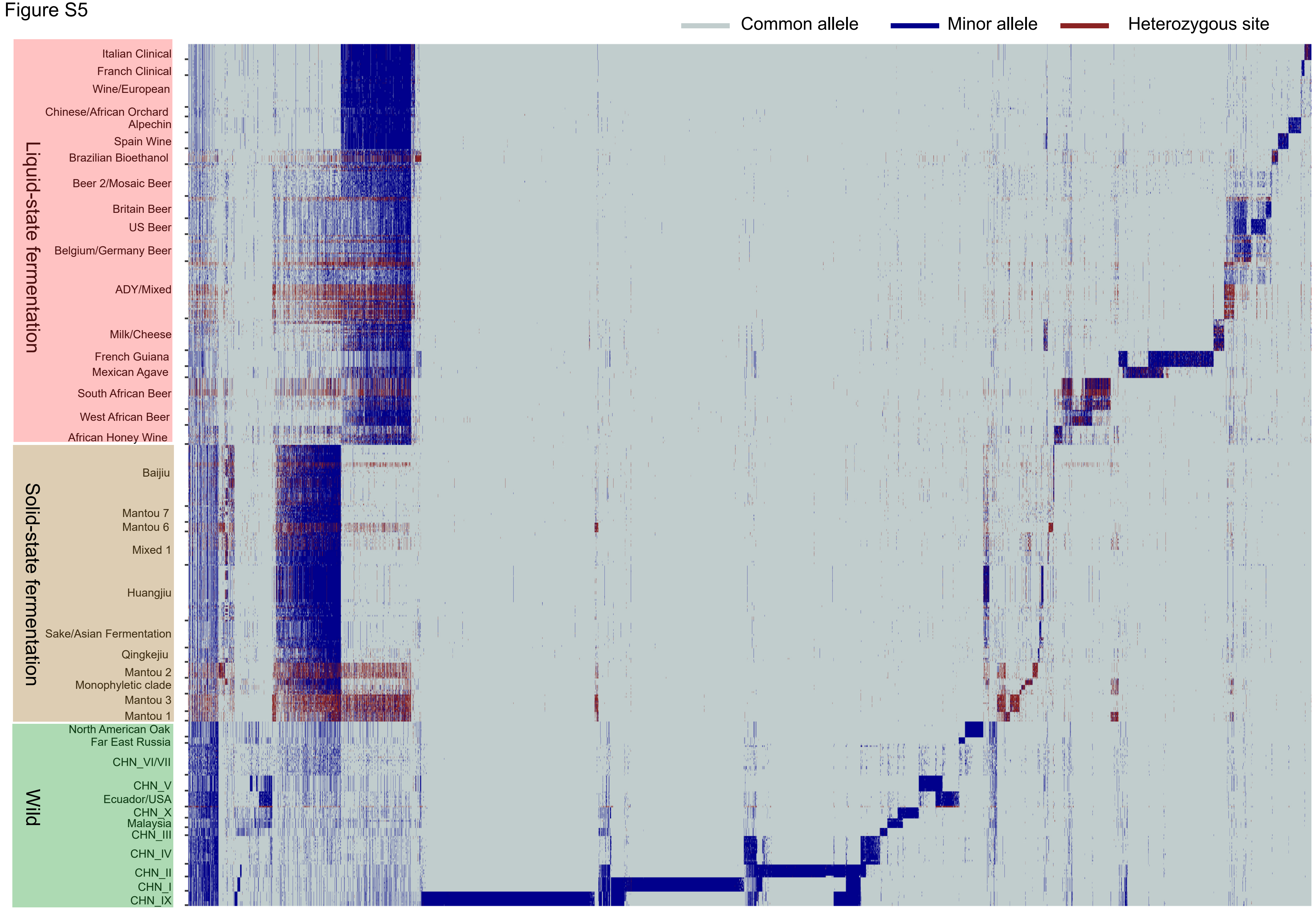

Distribution of 7,348 SNPs in 612 wild and domesticated S. cerevisiae isolates with worldwide origins.
The SNPs are filter from 10,000 randomly selected SNPs by excluding the sites that exist in less than 80% isolates of any lineages.
